# Supplementary material for: Burden of cystic and alveolar echinococcosis in the Kyrgyz Republic
Source: PLoS Negl Trop Dis. 2026 Mar 23;20(3):e0014074. doi: 10.1371/journal.pntd.0014074 (PMC13038107; doi:10.1371/journal.pntd.0014074)
Supplement: Attachment — Submitted filename: PNTD-D-25-01461_R1_reviewer.pdf [file pntd.0014074.s004.pdf]

PLOS Neglected Tropical Diseases

BURDEN OF CYSTIC AND ALVEOLAR ECHINOCOCCOSIS IN THE KYRGYZ REPUBLIC

--Manuscript Draft--

**Manuscript Number:**	PNTD-D-25-01461R1
**Full Title:**	BURDEN OF CYSTIC AND ALVEOLAR ECHINOCOCCOSIS IN THE KYRGYZ REPUBLIC
**Short Title:**	Burden of echinococcosis in Kyrgyz Republic
**Article Type:**	Research Article
**Keywords:**	Cystic echinococcosis<br>Alveolar echinococcosis<br>Kyrgyz Republic<br>Disease burden<br>DALYs<br>Economic impact<br>Health economics<br>Zoonotic diseases<br>Recurrent cases<br>Preventive measures
**Abstract:**	The purpose of this study is to assess some aspects of the economic and social burden of cystic and alveolar echinococcosis in the Kyrgyz Republic. From 2015-2020 a total of 5568 cases of primary cystic echinococcosis (CE) were reported and a further 1008 recurrent cases. Over the same time period 880 primary cases of primary alveolar echinococcosis (AE) were reported and a further 343 recurrent cases. The estimated DALYs for CE was 7849, that is 1308 per annum or 19.5 per 100,000 per annum. For AE was there were 3809 DALYs, that is 634 per annum or 9.5 per 100,000 per annum. The proportion of fatal cases of AE was 3.6 times higher than from CE. The total economic damage in terms of purchasing power equivalent (PPE) from CE amounted to more than 10,5 million international US dollars. For AE it amounted to more than 5.1 million international US dollars. Most of the financial burden falls on the patients themselves and amounts to 68.4%, i.e. 1223 PPE dollars per case out of total spending 1615 PPE dollars per case. To reduce the social and economic burden of echinococcosis, it is necessary to timely identify the disease, prevent the development of complications and multiple lesions, and promote preventive chemotherapy where appropriate. A deep understanding of the etiology, epidemiology, clinical manifestations, and tactics of early diagnosis of various forms of echinococcosis is also important for timely detection, effective control, and, most importantly, the organization of preventive measures to combat this disease.
**Additional Information:**	
**Question**	**Response**
**Government Employee**<br><br>Are you or any of the contributing authors an employee of the United States government?	No - No authors are employees of the U.S. government.

Powered by Editorial Manager® and ProduXion Manager® from Aries Systems Corporation

Manuscripts authored by one or more US Government employees are not copyrighted, but are licensed under a CC0 Public Domain Dedication, which allows unlimited distribution and reuse of the article for any lawful purpose. This is a legal requirement for US Government employees.<br><br>This will be typeset if the manuscript is accepted for publication.	
**Financial Disclosure**<br><br>Enter a financial disclosure statement that describes the sources of funding for the work included in this submission. Review the submission guidelines for detailed requirements. View published research articles from *PLOS Neglected Tropical Diseases* for specific examples.<br><br>This statement is required for submission and **will appear in the published article** if the submission is accepted. Please make sure it is accurate.<br><br>**Funded studies**<br>Enter a statement with the following details:<br>• Initials of the authors who received each award<br>• Grant numbers awarded to each author<br>• The full name of each funder<br>• URL of each funder website<br>• Did the sponsors or funders play any role in the study design, data collection and analysis, decision to publish, or preparation of the manuscript?<br><br>Did you receive funding for this work?	Yes
Please add funding details.<br>as follow-up to "**Financial Disclosure**<br><br>Enter a financial disclosure statement that describes the sources of funding for the work included in this submission. Review	This work was supported by the Swiss National Science Foundation (SNSF, grant agreement number 173131—"Transmission modelling of emergent echinococcosis in Kyrgyzstan") to GM and to PRT. URL: https://www.snf.ch/en.<br>The funders did not play any role in the study design, data collection and analysis, preparation of the manuscript of decision to publish

the submission guidelines for detailed requirements. View published research articles from *PLOS Neglected Tropical Diseases* for specific examples.<br><br>This statement is required for submission and **will appear in the published article** if the submission is accepted. Please make sure it is accurate.<br><br>**Funded studies**<br>Enter a statement with the following details:<br>• Initials of the authors who received each award<br>• Grant numbers awarded to each author<br>• The full name of each funder<br>• URL of each funder website<br>• Did the sponsors or funders play any role in the study design, data collection and analysis, decision to publish, or preparation of the manuscript?<br><br>Did you receive funding for this work?"	
Please select the country of your main research funder (please select carefully as in some cases this is used in fee calculation).<br>as follow-up to "**Financial Disclosure**<br><br>Enter a financial disclosure statement that describes the sources of funding for the work included in this submission. Review the submission guidelines for detailed requirements. View published research articles from *PLOS Neglected Tropical Diseases* for specific examples.<br><br>This statement is required for submission and **will appear in the published article** if the submission is accepted. Please make sure it is accurate.	SWITZERLAND - CH

Powered by Editorial Manager® and ProduXion Manager® from Aries Systems Corporation

**Funded studies**<br>Enter a statement with the following details:<br>• Initials of the authors who received each award<br>• Grant numbers awarded to each author<br>• The full name of each funder<br>• URL of each funder website<br>• Did the sponsors or funders play any role in the study design, data collection and analysis, decision to publish, or preparation of the manuscript?<br><br>Did you receive funding for this work?"	
**Competing Interests**<br><br>On behalf of all authors, disclose any competing interests that could be perceived to bias this work.<br><br>This statement will be typeset if the manuscript is accepted for publication.<br><br>Review the instructions link below and PLOS NTDs' competing interests policy to determine what information must be disclosed at submission.	The authors have declared that no competing interests exist
**Data Availability**<br><br>Provide a **Data Availability Statement** in the box below. This statement should detail where the data used in this submission can be accessed. This statement will be typeset if the manuscript is accepted for publication.<br><br>Before publication, authors are required to make all data underlying their findings fully available, without restriction. Review our PLOS Data Policy page for detailed information on this policy. Instructions for writing your Data Availability statement can be accessed via the Instructions link below.	Some of the data is available in the manuscript. Sources of other data is referenced and available from the internet. Here the URL is given in the reference list (references 24-32)

Manuscript

Click here to access/download;Manuscript;MGA_revised_trackchangesremo

BURDEN OF CYSTIC AND ALVEOLAR ECHINOCOCCOSIS IN THE KYRGYZ REPUBLIC

Gulnara Minbaeva[1,2], Nelya Akhunbaeva[2], Kalis Nogoibaeva[2], Vera Toygombaeva [1,2], Kenesh Dzhusupov [1], Paul R. Torgerson[3]

[1]International Higher School of Medicine, Bishkek, Kyrgyzstan

[2]Kyrgyz State Medical Academy named after I.K. Akhunbaeva, Bishkek, Kyrgyzstan

[3]Section of Veterinary Epidemiology Zurich University, Switzerland

Abstract

The purpose of this study is to assess some aspects of the economic and social burden of cystic and alveolar echinococcosis in the Kyrgyz Republic. From 2015-2020 a total of 5568 cases of primary cystic echinococcosis (CE) were reported and a further 1008 recurrent cases. Over the same time period 880 primary cases of primary alveolar echinococcosis (AE) were reported and a further 343 recurrent cases. The estimated DALYs for CE was 7849, that is 1308 per annum or 19.5 per 100,000 per annum. For AE was there were 3809 DALYs, that is 634 per annum or 9.5 per 100,000 per annum. The proportion of fatal cases of AE was 3.6 times higher than from CE. The total economic damage in terms of purchasing power equivalent (PPE) from CE amounted to more than 10,5 million international US dollars. For AE it amounted to more than 5.1 million international US dollars. Most of the financial burden falls on the patients themselves and amounts to 68.4%, i.e. 1223 PPE dollars per case out of total spending 1615 PPE dollars per case. To reduce the social and economic burden of echinococcosis, it is necessary to timely identify the disease, prevent the development of complications and multiple lesions, and promote preventive chemotherapy where appropriate. A deep understanding of the etiology, epidemiology, clinical manifestations, and tactics of early diagnosis of various forms of echinococcosis is also important for timely detection, effective control, and, most importantly, the organization of preventive measures to combat this disease.

Introduction

Echinococcosis is a highly pathogenic parasitic diseases in humans, characterized by space occupying lesions in the liver, lungs and other organs, which often lead to disability and occasional mortality [1]. A study from Armenia reported a case fatality rate of 1.29% [2]. This disease can cause substantial economic damage due to ill health. Globally, the WHO Reference Group on the Burden of Foodborne Disease Burden of Diseases estimated that echinococcosis is responsible for 19,300 deaths and approximately 871,000 disability-adjusted life years lost each year [3].

Cystic echinococcosis (CE) is a zoonotic parasitic disease with a global distribution caused by the larval stage of *Echinococcus granulosus sensu lato*. The distribution of *E. granulosus* is considered worldwide, with only a few areas such as Iceland, Ireland, and New Zealand believed to be free of autochthonous human CE. In the former Soviet Union and Eastern Europe, there was an initial increase in the number of observed cases from 1991 [4]. For example in Bulgaria the reported incidence increased from 2 per 100,000 in 1991 to 8 per 100,000 in 1996. The incidence started to decline in the first decade of this century, and by 2013 was 4 cases per 100,000 per year [5,6]. Likewise in Kazakhstan, the incidence of CE increased from 1.4 cases per 100,000 in 1991 to 5.9 cases per 100,000 in 2000 [7]. Thereafter, the incidence has been between 4.7 and 5,6 cases per 100,000 per year [8]. At least 270 million people (58% of the total population) are at risk of CE in Central Asia including areas of Mongolia, Kazakhstan, Kyrgyzstan, Tajikistan, Turkmenistan, Uzbekistan, Afghanistan, Iran, Pakistan and western China. The annual surgical incidence rate in part of central Asia has been estimated to be as high as 25-27 cases per 100,000 in some districts [9-11] with prevalences reaching 10% (range from 0.8 to 11.9%) documented in some Tibetan communities in western China [12] and for Kyrgyzstan by Paternoster et al. [14] CE not may cause severe disease and possible death in humans, but also results in economic losses from treatment costs, lost wages, and livestock-associated

production losses. Globally, the annual cost of treating patients and losses in livestock production due to CE has been estimated at US$3 billion [1]

Alveolar echinococcosis (AE), caused by the larval stage of *Echinococcus multilocularis*, is confined to the northern hemisphere, in particular to North America, Central and Eastern Europe, Russia, Central Asia, Western China and Japan [13]. In humans, AE causes an infiltrating lesion primarily in the liver but in advanced cases metastasizing to other organs. It is usually a fatal disease if it remains untreated [14]. In some high endemic countries, the local prevalence of AE has been reported to be as high as 15% in some 7 villages in China [15] and up to 4% locally in Kyrgyzstan [16]. From 2014 to 2016, the surgical incidence of AE on a country-level in Kyrgyzstan was 3.02 per 100,000 inhabitants per year, which is considered to be the highest country-wide incidence for AE worldwide [17]. Globally, approximately 10,500 new AE cases occur every year [13]. In most parts of the world, AE is considered as an emerging disease with increasing incidences over the last decades, although there appears to be a downward trend in China where the majority of cases are reported [13]. In Europe, a few hundred AE cases are diagnosed in every year with an increasing trend. In Switzerland, the yearly overall incidence of AE has significantly increased from 0.1 per 100,000 inhabitants between 1993-2000 to 0.26 per 100,000 in 2001-2005 [18]. Recent data suggests the incidence has increased further to an annual incidence of over 0.58 cases per 100,000 in 2021-2022 [19]. Kyrgyzstan is a lower middle income, mountainous country with 48% of the population employed in agriculture and one third of the population living below the poverty line. In recent years there is increasing evidence that AE and CE are becoming an increasing public health problem in central Asia, particularly in Kyrgyzstan where hundreds of cases are reported annually [171,194]. The aims of this study were to estimate the burden of these disease in Kyrgyzstan and their monetary impact.

The global burden of AE and CE has been previously reported as approximately 690,000 and 184,00 DALYs respectively [3]. In Kyrgyzstan it has been previously estimated that the burden of AE is approximately 12,000 DALYs per annum or 50 DALYs per case. This assumed a case fatality rate approaching 100%. That for CE was approximately 3000 DALYs per annum, or 1.3 DALYs per cases with an assumed case fatality rate of 1% [20]. Treatment costs are highly variable depending on the local costs of medical treatment. For example, in Jordan, a lower middle income country, treatment costs for a case of CE was estimated at approximately US$500 in 2001 [21]. In contrast, in the United Kingdom, an upper income country the treatment costs of a single case of CE was estimated to be £7300 (aproximately US$10,000), also in 2001 [22]. Treatment costs of AE are higher and less well documented. In Switzerland, an upper income country, it was estimated that a single case of AE resulted in treatment costs of over US$100,000 [23]

Methods

Ethics

Ethical approval for the study was granted by the ethics committee of the Ministry of Health in Kyrgyzstan.

Patient data

The patient data were retrieved from the database of the “Automated Information System for Surveillance of Infectious and Parasitic Diseases” (AIS) of the Department of Disease Prevention and Public Health Surveillance of the Ministry of Health of the Kyrgyz Republic for 2015-2020 [24] . Among others, relevant variables extracted for this study included date and clinical presentation of CE/AE, all surgical and anti-helminthic treatments in the in- and outpatient setting and long-term disability due to CE/AE or its treatment.

Data on the incidence and fatalities for CE and AE were analyzed from the AIS database for 2015-2020. Both diseases were cases based on clinical, pathological and histological confirmation of the lesion. Relapsed cases were those with the same diagnosis of CE or AE at the same anatomical site six or more months after discharge following treatment. Case reports included individual residential address, sex, age, occupation, diagnosis, i.e., CE, AE, CE relapse, or AE relapse. The collected data were summed into the predefined 5 year age- and sex-categories, i.e. 0-4 yr, 5-9 yr, 10-14 yr, 15-19 yr, 20-24 yr, 25-29 yr, 30-34 yr, 35-39 yr, 40-44yr, 45-49yr, 50-54yr, 55-59yr, 60-64yr, 65-69 yr, 70-74 yr, 75-79 yr, 80-84yr, >85 separately for males and females.

Disability-adjusted life years (DALY) estimates

The disability-adjusted life year in its simplest terms can be considered a loss of healthy years of life and is a non-monetary measure of disease burden. It consists of two components: years lived with disability (YLDs) and years of life lost (YLLs). YLDs were calculated according to standard methods [3] using an incidence based approach for YLDs For patients diagnosed with echinococcosis a disability weight of 0.2, the same as for liver cancer (Dutch disability weight for disease free liver cancer) was assigned [1]. This disability weight was assumed for the length of the follow-up period. The WHO life table [25] was used to estimate YLLs for fatal cases

Financial estimates

To assess the economic burden of CE and AE, an analysis of direct financial expenses of the state reimbursed by the Compulsory Medical Insurance Fund (CMIF). The data were officially presented by the CMIF of the Kyrgyz Republic, the cost of expenditure was determined in Kyrgyzstani som- (KGS). Additional expenses of the patients themselves during inpatient treatment of patients for 2015-2020 was carried out. Such costs include

anti-relapse therapy, additional instrumental and laboratory tests and additional expenses of the patients themselves during the rehabilitation period which are not covered by the Medical Insurance Fund. Costs are presented in KGS, US $ and purchasing power equivalent $ (PPE), that is international dollars. The latter reflects the costs of goods and services in Kyrgyztsan compared to the costs of the same goods and services in the USA. Such goods and services are generally cheaper in Kyrgyzstan compared to the USA, thus $3.94 worth of goods and services in the USA would cost just $1 in Kyrgyzstan. Thus the international dollar exchange rate used was 21.74 som to 1 US $ (2022 rate)[26]. The actual exchange rate used was 85.50 som to the US $, also the 2022 exchange rate (end of year)[27]. The costs of computer tomography, ultrasound examination, serological studies, histology and chemotherapy (albendazole) were sourced from [28-32]

Statistical analysis

Comparisons of proportions was undertaken by the Fisher exact test whilst exact binomial confidence intervals were reported. All calculations were done in R [33].

Results

The incidence of CE and AE from 2002 to 2022 among the population of the Kyrgyz Republic presented in Figure 1.

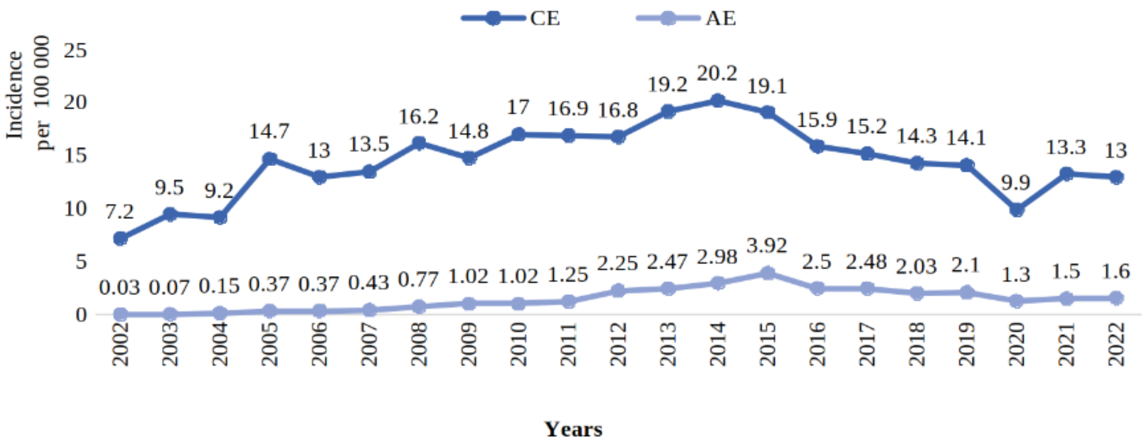

Figure 1. The incidence of new cases CE and AE among the population of the Kyrgyz Republic, 2001-2022. Incidence calculated from the numbers of cases notified to the Ministry of Health.

The data in Table 1. show that for the period 2015-2020 7,799 cases of echinococcosis received hospital treatment. For CE the proportion of cases that were primary cases was 84.7% on average with recurrent cases accounting for the remaining 15.3% of cases. The proportion of recurrent cases for AE was 28.0% . Of the total number of cases undergoing surgical treatment, the proportion of recurrences in AE was 1.8 times higher than in CE ($p<0.05$).

Compared to 2015, there has been an increase in the incidence of recurrent cases rate since 2016, with a peak in 2018 of 4.4 per 100,000 and a subsequent downward trend by 2020 to 1.7 per 100,000.

An analysis of the reported cases of a 6-year period as a whole showed that for the period 2015-2020 mortality in AE was 3.6 times higher than for CE , For AE 36 cases from 1,223 registered cases had a fatal outcome (2.9%) whilst for CE there were where 55 fatalities from 6,576 registered cases 0.9% ($p<0.0001$).

For children under 14 years of age there were three fatal cases of AE from 30 registered case (10%), which was not significantly different from the adult case fatality rate of 33 fatal cases from 1,193 registered cases ($p=0.055$). For CE there were no fatal cases reported among the 1,136 registered pediatric cases, compared to 55 deaths from the 4,940 registered cases in adults ($p<0.0001$, Tables 1 and 2).

Table 1. The ratio of patients with primary n=6,448 and recurrent n=1,351 CE and AE treated in hospitals, (n=7,799) and the number of fatal cases (n=91), Kyrgyz Republic, 2015-2020.

	Cystic echinococcosis		
	Primary	Recurrent	Proportion Recurrent (95% CI)
2015	1,134	34	2.9% (2.0% to 4.0%)
2016	963	213	21.1% (19.5% to 22.6%)
2017	942	225	22.3% (20.7% to 23.8%)
2018	906	250	24.8% (23.2% to 26.3%)
2019	970	194	19.2% (17.6% to 20.7%)
2020	653	92	9.1% (7.5% to 10.6%)
**Total**	**5,568**	**1,008**	**15.3% (13,7% to 16,8%)**
	Alveolar echinococcosis		
	Primary	Recurrent	Proportion Recurrent (95% CI)
2015	235	87	25.4% (24.0% to 26.7%)
2016	150	53	15.5% (14.1% to 16.8)
2017	149	73	21.3% (19.9% to 22.6%)
2018	121	66	19.2% (17.8% to 20.5%)
2019	137	42	12.2% (10.8% to 13.5%)
2020	88	22	6.4% (5.0% to 7.7%)
**Total**	**880**	**343**	**28.0% (26.6% to 29.3%)**

Table 2. Case fatality rates of CE and AE cases treated in hospitals.

	Fatal cases from CE			Fatal cases from AE		
	Number of cases	Fatalities	Case fatality rate (95% CI)	n		95% CI
2015	1,168	8	0.7% (0.3% to 1.4%)	322	5	1.5% (0.5% to 3.5%)
2016	1,176	5	0.4% (0.1% to 1.0%)	203	3	1.4% (0.03% to 4.2%)
2017	1,167	13	1.1% (0 .5% to 1.9%)	122	7	3.1% (1.2% to 6.3%)
2018	1,156	14	1.2% (0.6% to 2.0%)	187	10	5.3% (2.5% to 9.6%)
2019	1,166	11	0.9% (0.4% to 1.6%)	179	7	3.9% (1.5% to 7.8%)
2020	745	4	0.5% (0.1% to 1.3%)	110	4	3.6% (1.0% to 9.0%)
**Total**	**6,576**	55	0.8% (0.6% to 1.0%)	1,223	36	2.9% (2.0% to 4.0%)

The total DALY from CE was 7,849 and from AE was 3,809 over 6 years (Table 3) which is a mean of 1,308 and 634 DALYs respectively per year. Each deceased patient with CE lost a mean of 48 YLLs and whilst fatal AE cases lost a mean of 46

YLLs. The annual burden of disease per 100,000 population for CE was 19.5 DALYs and that for AE was 9.5 DALYs. For CE, it was estimated that there was approximately 1.2 DALYs per case, whilst for AE it was approximately 3.1 DALYs per case.

Table 3 presents the age stratified DALYs estimated during the study period. This includes the relative contributions from YLLs and YLDs. As can be seen from the data in the Kyrgyz Republic, the CMIF for 2015-2020 reimbursed financial expenses to healthcare organizations for inpatient treatment with primary and recurrent CE and AE for 7,799 patients. In general, over five years, the total cost of treatment with additional expenses for patients amounted to ≈ 66.9 million som (KGS), that is 782,711 US dollars.

The average cost of one treated (direct state costs) case of CE is 8,376 KGS and alveolar echinococcosis is 9,680 KGS. The average cost of CE and AE was 8,580 KGS per patient, that is, ≈100 US dollars. When converted to the international exchange rate against the US dollar, which was 21.74 at the time of calculation (https://en.wikipedia.org/wiki/International_dollar), the cost to the government per patient would be the purchasing power equivalent (PPE) of 394 US dollar (Table 4).

The total financial costs for CE amounted to 55 082 046 million KGS or 644,234 US $. Direct government expenditures for the treatment of AE amounted to 11,839,735 million KGS or 138 476 US $.

As shown in Table 5, the direct costs of the patients themselves, in comparison with the direct costs of the state, are three times higher. For CE the state spends a total of $98 per patient and the patients' own costs are $311, which is 3.2 times more. For alveolar echinococcosis, the state spends a total of $100 per patient, and patients' own costs are $420, which is 4.2 times more. According to our calculations, about 73% of the costs are borne by the patients themselves, which has a direct negative impact on the economic situation of the family. It should be noted that our calculations do not take into account indirect costs such as transportation, additional food, patient care, accommodation and temporary disability.

Table 3. Age distribution of fatal cases of CE and AE and the DALYs estimate of reduction in life expectancy from due to premature death (AE), Kyrgyz Republic, 2015-2020.

Age	Residual life expectance	Cases n=6,576	Deaths n=55	YLLs	YLDs fatal	YLDs non fatal	DALYs non fatal	DALYs fatal	Total DALY
	**Cystic Echinococcosis**								
0-4	87.1	103	0	0.0	0.0	9.4	9.4	0.0	9.4
5-9	82.6	223	0	0.0	0.0	81.4	81.4	0.0	81.4
10-14	77.6	810	0	0.0	0.0	138.5	138.5	0.0	138.5
15-19	72.6	342	8	580.8	2.5	102.8	686.1	581.2	1269.3
20-24	67.6	37	5	338.1	0.4	11.7	350.1	339.0	690.1
25-29	62.7	23	1	62.7	0.3	7.3	70.3	63.6	133.3
30-34	57.7	458	7	404.0	0.5	205.7	610.1	404.9	1017.2
35-39	52.8	359	5	263.8	0.4	157.4	421.6	264.7	687.7
40-44	47.8	521	3	143.5	0.5	236.2	380.2	144.6	525.0
45-49	42.9	1,312	5	214.7	0.5	596.0	811.1	215.7	1028.1
50-54	38.1	752	8	304.8	.4	322.3	627.5	306.4	935.8
55-59		784	3	100.0	0.5	356.1	456.6	101.0	557.9
60-64	28.7	300	3	86.0	0.4	132.0	218.5	88.3	305.8
65-69	24.1	456	2	48.2	0.4	170.8	219.4	48.8	268.4
70-74	19.8	58	0	0.0	0.0	26.4	26.4	0.0	26.4
75-79	15.7	38	5	78.3	0.5	15.0	93.8	78.5	174.3
80-84	11.96	0	0	0	0	0	0	0.0	0.0
85<	7.05	0	0	0	0	0	0	0.0	0.0
**Total**	**811.0**	**6,576.0**	**55**	**2,624.8**	**7.1**	**2,569.0**	**5,200.9**	**2,636.5**	**7,849**
	**Alveolar Echinococcosis**								
Age	Residual Life Expectancy	AE cases n=1223	AE deaths n=36	YLL AE	YLDs fatal AE	YLDs non fatal AE	DALYs non fatal AE	DALYs fatal	Total DALY
0-4	87.1	1	1	87.1	0.2	0.0	87.2	87.2	174.5
5-9	82.6	29	2	165.2	0.6	8.0	173.8	165.8	339.5
10-14	77.6	43	0	0.0	0.0	60.8	60.8	0.0	60.8
15-19	72.6	36	1	72.6	0.3	11.2	84.1	72.9	157.0
20-24	67.6	57	0	0.0	0.0	14.3	14.3	0.0	14.3
25-29	62.7	172	1	62.7	0.3	58.5	121.5	63.0	184.5
30-34	57.7	112	4	230.8	1.1	30.8	262.8	232.0	494.7
35-39	52.8	200	9	474.8	4.0	84.9	563.8	478.8	1042.6
40-44	47.8	146	1	47.8	0.3	44.6	92.8	48.1	140.9
45-49	42.9	252	2	85.9	0.8	105.5	192.2	86.7	278.9
50-54	38.1	86	2	76.2	0.7	29.7	106.6	76.9	183.5
55-59	33.3	28	2	66.7	0.7	9.5	76.9	67.4	144.3
60-64	28.7	31	7	200.6	1.8	6.0	208.4	202.4	410.8
65-69	24.1	0	2	48.2	0.0	0.0	48.2	48.2	96.5
70-74	19.8	13	2	39.5	0.4	2.4	42.3	40.0	82.3
75-79	15.7	17	0	0.0	0.0	3.7	3.7	0	3.7
80-84	11.96	0.0	0.0	0.0	0.0	0.0	0.0	0.0	0.0
85<	7.05	0.0	0.0	0.0	0.0	0.0	0.0	0.0	0.0
**Total**	**811.0**	**1,223.0**	**36.0**	**1,658.1**	**11.3**	**469.8**	**2,139.2**	**1,669.4**	**3,809**

Table 4 Direct government costs (in KGS) for the treatment of patients with various forms of CE, AE and unspecified diagnoses in hospitals across the country, 2015-2020. Data from the health insurance fund.

Cystic echinococcosis							
Site of primary lesion	Per Case				Total Costs		
	KGS	Exchange rate 85.50	Internationao doller pp exchange rate = 21.74	number of cases	KGS	Exchange rate 85.50	Internationaol doller pp exchange rate = 21.74
Liver	7,108	83	327	1,620	11,514,960	134,678	529,667
Lung	9,104	106	419	360	3,277,440	38,333	150,756
Bone	7,211	84	332	120	865,320	10,121	39,803
Other local./multip.	8,483	99	390	2,730	23,158,590	270,861	1,065 253
Unspecified	9,316	109	429	1,746	16,265,736	190,243	748,194
**Total CE**	**8,376**	**98**	**385**	**6,576**	**55,082,046**	**644,234**	**2,533,673**
Alveolar echinococcosis							
Site of primary lesion	KGS	Exchange rate 85.50	Internationao doller pp exchange rate = 21.74	n	KGS	Exchange rate 85.50	Internationaol doller pp exchange rate = 21.74
Liver	8,121	95	374	241	1 ,957,161	22,891	90,026
Other local./multip.	10,123	118	466	940	9,515,620	111,294	437,701
Unspecified	8,737	102	402	42	366,954	4 292	16,879
**Total AE**	**9,680**	**113**	**445**	**1,223**	**11 839 735**	**138 476**	**544,606**
**Total government cost**	**8,580**	**100**	**394**	**7,799**	**66921,781**	**782,711**	**3,078,279**

Table 5 Additional Direct costs of patients (in KGS) with various forms of diagnoses CE and AE in hospitals across the country, n=3619, 2015-2020.

	CE n=1	CE N=6,576	AE n=1	AE N= 1,223	Total N= 7,799
Complete blood count, complete urine test, biochemical tests (creatinine, bilirubin, blood clotting), electrocardiography	2,000	13,152,000	2,000	2,446,000	15,598,000
Additional expenses: Computed tomography, ultrasound, serological studies	5,950	39,127,200	11,250	13,758,750	68,558,050
Blood plasma 1 dose	0	0	1,700	2,079,100	7,106,000
Histological analyzes	1,990	13,086,240	1,990	2,433,770	15,520,010
Antibiotic resistance tests	1,368	8 995 968	1,368	1,673,064	10,669,032
Cost for surgery	11,300	74,308,800	13,600	16,632,800	97,742 700
Costs for 3 months of anti-relapse chemotherapy (drug Albendazole)	3,982	26,185,632	3,982	4,869,986	31,055,618
**Total cost patients**	**26,590**	**174,855,840**	**35,890**	**43,893,470**	**246,249,410**
**Exchange rate 85.50**	**311**	**2,045,098**	**420**	**513,374**	**2,880,110**
**International dollar pp exchange rate = 21.74**	**1,223**	**8,043,047**	**1,651**	**2,019,019**	**11,327,020**

The total economic damage in terms of PPE from CE should have amounted to more than 10.5 million international US dollars. For AE should have amounted to more than 5.1 million US dollars. The total economic damage in terms of PPE from CE and AE should have amounted to more than 14.4 US million international dollars. Most of the financial burden falls on the patients themselves and amounts to 68.4%, i.e. 1223 PPE dollars out of total spending 1615 PPE dollars per case.

Discussion

The World Health Organization (WHO) has included CE in its official list of 18 neglected tropical diseases. In addition to this group, WHO has defined CE as belonging to a subgroup of seven endemic or “neglected zoonotic diseases”. Echinococcosis is common where there is poverty and a dependence on livestock. These are indicators of the quality of

social and financial capital, low resilience and close proximity of people and their animals contribute to the transmission of this disease [34]. In the context of echinococcosis surveillance and control, mass screening surveys provide valuable data that will help reduce the medical, social and economic burden by ensuring early detection and rapid treatment of cases and in humans.

There was a substantial decrease in the number of reported cases and deaths due to CE and AE in 2020, which is associated with the outbreak of Coronavirus. An increase in the number of deaths from echinococcosis was observed in 2017, 2018 and 2019. The case fatality rate of 2.9%, is higher for AE compared to the 0.8% for CE AE would usually have a high case fatality rate, although concomitant treatment with albendazole following liver resection does improve the prognosis [14,35]. The low case fatality rate reported in the present study may be due to the under reporting of deaths with AE, which could be registered as liver failure or neoplasia. Also, the large increases in the reported cases of AE over the last 20 years indicate an evolving epidemic. AE is a chronic disease and death may occur several years after diagnosis, so AE cases reported in the present study may result in fatalities in the future. Alternatively, with increasing use of albendazole following surgical treatment may be dramatically decreasing the case fatality rate.

The sharp rise in treatment costs—particularly by 1.4 times noted in the year 2020 compared to 2017—reflects both increased awareness and reporting of the disease as well as potentially rising prices for medical care and pharmaceuticals. It should be noted that in the sum of CE and AE diseases, the share of costs for the treatment of AE of other organs unspecified more than 9.5 million KGS, i.e. ≈111.3 thousand US$. However, the clinical manifestations of CE are more diverse, and the economic burden of cases with multiple localization more than 23 million KGS, i.e. 270 thousand US$. This indicates a late detection of this form of clinical manifestation of CE.

The high number of disability-adjusted life years – DALYs deaths demonstrates not only the deadliness of these diseases. There will be an additional social and economic burden on families and society as a whole. Furthermore, many cases, especially those in remote areas where there is poor medical services infrastructure, may go undiagnosed and unreported. For example, in the remote Alay valley in Kyrgyzstan, a prevalence of over 4% for AE in the population has been reported [16]. Such unreported cases will add significantly to the burden of disease. The DALY estimated in the present report are somewhat lower for AE than previously estimated by Coulotte et al. [20]. This is because in the previous study, AE was assumed to have a high case fatality rate and estimated YLLs on the assumption that most diagnosed cases of AE would eventually be fatal cases. The present study estimates the YLLs only on reported fatal cases and will be an underestimate if a substantial number of the AE cases would indeed be fatal, either due to misdiagnosis or deferred deaths at a later date. The burden of disease of CE of 1.2 DALYs per case is in line with previous studies [3], but the 3.1 DALYs per case for AE is somewhat lower than other studies and is likely to be due to the reported case fatality rate as described above.

The annual incidence of CE in Kyrgyzstan, based on hospital records, increased from 5.4 cases/100,000 in 1991 to 14 cases/100,000 in 1998 [10], and then to 20 cases/100,000 in 2014 (the present study). Between 1995 and 2011, human AE increased from <3 cases per year to >60 cases per year and then to over 200 per year by 2015 [17, 36]. The prevalence of CE also increased in major livestock species such as sheep with a doubling of reported prevalence in some areas [10].

Public Health Challenges

Echinococcosis is emblematic of larger public health challenges facing the Kyrgyz Republic, particularly the integration of effective zoonotic disease management with overall health system strengthening. The persistence and escalation of echinococcosis

burden underscores the need for a One Health approach, which links human, animal, and environmental health strategies cohesively.

The increasing economic and social burden of CE and AE highlights the need for enhanced public health policy interventions in the Kyrgyz Republic. Enhanced funding for health services, especially in rural and under-served areas, is critical. Implementing widespread screening and early diagnosis programs, particularly in high-risk areas, could significantly reduce the incidence and severity of the disease. Public health campaigns aimed at educating the population about the transmission, dog population management and prevention of echinococcosis can also play a crucial role. Additionally, strengthening the healthcare infrastructure to better manage and treat cases will mitigate the long-term economic impact and improve patient outcomes.

Limitations of the study.

One of the main limitations of the study is the potential under-reporting of cases and deaths associated with echinococcosis, which may lead to an underestimation of the disease's true burden. The study authors may miss unreported cases, especially in rural areas where there is a lack of medical reporting and resources, as well as AE deaths may be reported as cancer, cirrhosis, etc. Furthermore, the economic estimates do not account for indirect costs such as lost productivity and long-term disability, which could significantly increase the calculated burden. Furthermore, the monetary value of deaths has not been estimated as this is a controversial area in health economics and was one of the reasons the DALY was adopted to assess the burden of disease.

Future Research Perspectives

Future studies should aim to incorporate factors mentioned above to provide a more comprehensive view of the burden of echinococcosis. Such research should focus on

improving disease surveillance and reporting mechanisms to capture all cases and outcomes related to echinococcosis, thus providing a more accurate and comprehensive data set.

Further research is necessary to explore the genetic and molecular aspects of *Echinococcus* species prevalent in the region to understand the transmission dynamics better and develop targeted treatments. Additionally, studies assessing the impact of climate change on the epidemiology of echinococcosis could provide insights into future trends and help in formulating adaptive strategies.

Conclusion

This comprehensive analysis of CE and AE in the Kyrgyz Republic has underscored a significant escalation in both the economic and social burdens of these diseases over the period studied. Our findings illuminate the growing incidence of these conditions and their profound impact on public health systems, individual patients, and broader societal dynamics.

Our study calls for a robust response to echinococcosis due to substantial costs and disease burden. This should emphasize the importance of sustained financial investment in health infrastructure, the enhancement of epidemiological surveillance, and the mobilization of community education efforts. By addressing echinococcosis with a comprehensive and proactive approach, the Kyrgyz Republic can better manage and eventually reduce the burden of this severe disease, leading to improved public health outcomes and enhanced economic stability.

Conflict of interest. We have no financial interest or other benefits from the publication of this article.

Acknowledgments. This work was supported by the Swiss National Science Foundation (SNSF, grant agreement number 173131- "Transmission modelling of emergent echinococcosis in Kyrgyzstan"). We also wish to acknowledge the the MHIF staff who provided data from the reporting forms.

References

1. Budke CM, Deplazes P, Torgerson PR. Global socioeconomic impact of cystic echinococcosis. Emerg Infect Dis. 2006;12: 296–303.

2. Khachatryan, Anna S. Analysis of lethality in echinococcal disease. *The Korean journal of Parasitology*. 2017*;* 55: 549-553

3.Torgerson PR, Devleesschauwer B, Praet N, Speybroeck N, Willingham AL, Kasuga F, et al. World Health Organization Estimates of the Global and Regional Disease Burden of 11 Foodborne Parasitic Diseases, 2010: A Data Synthesis. PLoS Med. 2015;12: e1001920. doi: 10.1371/journal.pmed.1001920

4. Torgerson PR. The emergence of echinococcosis in central Asia. Parasitology. 2013;140. doi:10.1017/S0031182013000516

5. Todorov, T. and Boeva, V., 1999. Human echinococcosis in Bulgaria: a comparative epidemiological analysis. *Bulletin of the World Health Organization*, *77*(2), p.110.

6. Jordanova, D.P., Harizanov, R.N., Kaftandjiev, I.T., Rainova, I.G. and Kantardjiev, T.V., 2015. Cystic echinococcosis in Bulgaria 1996–2013, with emphasis on childhood infections. *European Journal of Clinical Microbiology & Infectious Diseases*, *34*(7), pp.1423-1428.

7. Torgerson, P.R., Shaikenov, B.S., Baitursinov, K.K. and Abdybekova, A.M., 2002. The emerging epidemic of echinococcosis in Kazakhstan. *Transactions of the Royal Society of Tropical Medicine and Hygiene*, *96*(2), pp.124-128.

8. Mustapayeva, A., Manciulli, T., Zholdybay, Z., Juskiewicz, K., Zhakenova, Z., Shapiyeva, Z., Medetov, Z., Vola, A., Mariconti, M., Brunetti, E. and Budke, C.M., 2019. Incidence rates of surgically managed cystic echinococcosis in Kazakhstan, 2007–2016. *The American journal of tropical medicine and hygiene*, *102*(1), p.90.

9. Babadjanov AK, Yakubov FR, Ruzmatov PY, Sapaev DS. Epidemiological aspects of echinococcosis of the liver and other organs in the Republic of Uzbekistan. Parasite Epidemiol Control. 2021;15: e00230. doi: 10.1016/j.parepi. 2021.e00230

10. Torgerson PR, Oguljahan B, Muminov AE, Karaeva RR, Kuttubaev OT, Aminjanov M, et al. Present situation of cystic echinococcosis in Central Asia. Parasitol Int. 2006;55 Suppl: S207-212. doi: 10.1016/j.parint.2005.11.032

11. Zhang W, Zhang Z, Wu W, Shi B, Li J, Zhou X, et al. Epidemiology and control of echinococcosis in central Asia, with particular reference to the People's Republic of China. Acta Trop. 2015;141: 235–243. doi: 10.1016/j.actatropica.2014.03.014

12.Li, T., Chen, X., Zhen, R., Qiu, J., Qiu, D., Xiao, N., Ito, A., Wang, H., Giraudoux, P., Sako, Y. and Nakao, M., 2010. Widespread co-endemicity of human cystic and alveolar echinococcosis on the eastern Tibetan Plateau, northwest Sichuan/southeast Qinghai, China. *Acta tropica*, *113*(3), pp.248-256.

13. Lundström-Stadelmann B, Rostami A, Frey CF, Torgerson PR, Riahi SM, Bagheri K, et al. Human alveolar echinococcosis–global, regional, and national annual incidence and prevalence rates. Clin Microbiol Infect. 2025 [cited 15 Apr 2025]. doi: 10.1016/j.cmi.2025.01.034

14. Torgerson PR, Schweiger A, Deplazes P, Pohar M, Reichen J, Ammann RW, et al. Alveolar echinococcosis: from a deadly disease to a well-controlled infection. Relative survival and economic analysis in Switzerland over the last 35 years. J Hepatol. 2008;49: 72–77. doi: 10.1016/j.jhep.2008.03.023

15. Craig, P. S., Giraudoux, P., Shi, D., Bartholomot, B., Barnish, G., Delattre, P., & Vuitton, D. A. (2000). An epidemiological and ecological study of human alveolar echinococcosis transmission in south Gansu, China. *Acta tropica*, *77*(2), 167-177.

16. Bebezov B, Mamashev N, Umetaliev T, Ziadinov I, Craig PS, Joekel DE, et al. Intense Focus of Alveolar Echinococcosis, South Kyrgyzstan. Emerg Infect Dis. 2018;24: 1119–1122. doi:10.3201/eid2406.161641

17. Paternoster G, Boo G, Wang C, Minbaeva G, Usubalieva J, Raimkulov KM, et al. Epidemic cystic and alveolar echinococcosis in Kyrgyzstan: an analysis of national surveillance data. Lancet Glob Health. 2020;8: e603–e611. doi:10.1016/S2214-109X (20)30038-3

18. Schweiger A, Ammann RW, Candinas D, Clavien P-A, Eckert J, Gottstein B, et al. Human alveolar echinococcosis after fox population increase, Switzerland. Emerg Infect Dis. 2007;13: 878–882. doi:10.3201/eid1306.061074

19. Bresson-Hadni, S. For the Swiss Echinococcosis Network (2025) Alveolar echinococcosis in Switzerland. Leading Opinions Médecine Interne 1-2/2023 Available: https://www.universimed.com/ch/article/gastroenterologie/alveolar-switzerland-265336

20. Counotte MJ, Minbaeva G, Usubalieva J, Abdykerimov K, Torgerson PR. The Burden of Zoonoses in Kyrgyzstan: A Systematic Review. PLoS Negl Trop Dis. 2016;10: e0004831. doi: 10.1371/journal.pntd.0004831

21.Torgerson, P. R., Dowling, P. M., & Abo-Shehada, M. N. (2001). Estimating the economic effects of cystic echinococcosis. Part 3: Jordan, a developing country with lower-middle income. *Annals of Tropical Medicine & Parasitology*, *95*(6), 595-603.

22. Torgerson, P. R., & Dowling, P. M. (2001). Estimating the economic effects of cystic echinococcosis. Part 2: an endemic region in the United Kingdom, a wealthy, industrialized economy. *Annals of Tropical Medicine & Parasitology*, *95*(2), 177-185.

23. Torgerson, P. R., Schweiger, A., Deplazes, P., Pohar, M., Reichen, J., Ammann, R. W., et al. (2008). Alveolar echinococcosis: from a deadly disease to a well-controlled infection.

Relative survival and economic analysis in Switzerland over the last 35 years. *Journal of hepatology*, *49*(1), 72-77.
24. http://iepid.med.kg
25. WHO, A. (2020). WHO methods and data sources for life tables 1990–2019. *World Health Organization Geneva.* Available: https://cdn.who.int/media/docs/default-source/gho-documents/global-health-estimates/ghe2019_life-table-methods.pdf
26. https://en.wikipedia.org/wiki/International_dollar
27. https://tradingeconomics.com/kyrgyzstan/currency
28. https://cortex.kg/prices/
29. https://aqualab.kg/analyzes/
30. https://invitro.kg/analizes/
31. https://dgsen.kg
32. https://neman.kg
33. R Core Team (2024). R: A Language and Environment for Statistical Computing_. R Foundation for Statistical Computing, Vienna, Austria. <https://www.R-project.org/>.
34. Otero-Abad, B., & Torgerson, P. R. (2013). A systematic review of the epidemiology of echinococcosis in domestic and wild animals. *PLoS neglected tropical diseases*, *7*(6), e2249.
35. Brunetti E, Kern P, Vuitton DA. Expert consensus for the diagnosis and treatment of cystic and alveolar echinococcosis in humans. Acta Trop. 2010;114: 1–16. doi: 10.1016/j.actatropica.2009.11.001
36. Usubalieva, J., Minbaeva, G., Ziadinov, I., Deplazes, P., & Torgerson, P. R. (2013). Human alveolar echinococcosis in Kyrgyzstan. *Emerging Infectious Diseases*, *19*(7), 1095.

Figure 1

Click here to access/download;Figure;Figure 1.tif

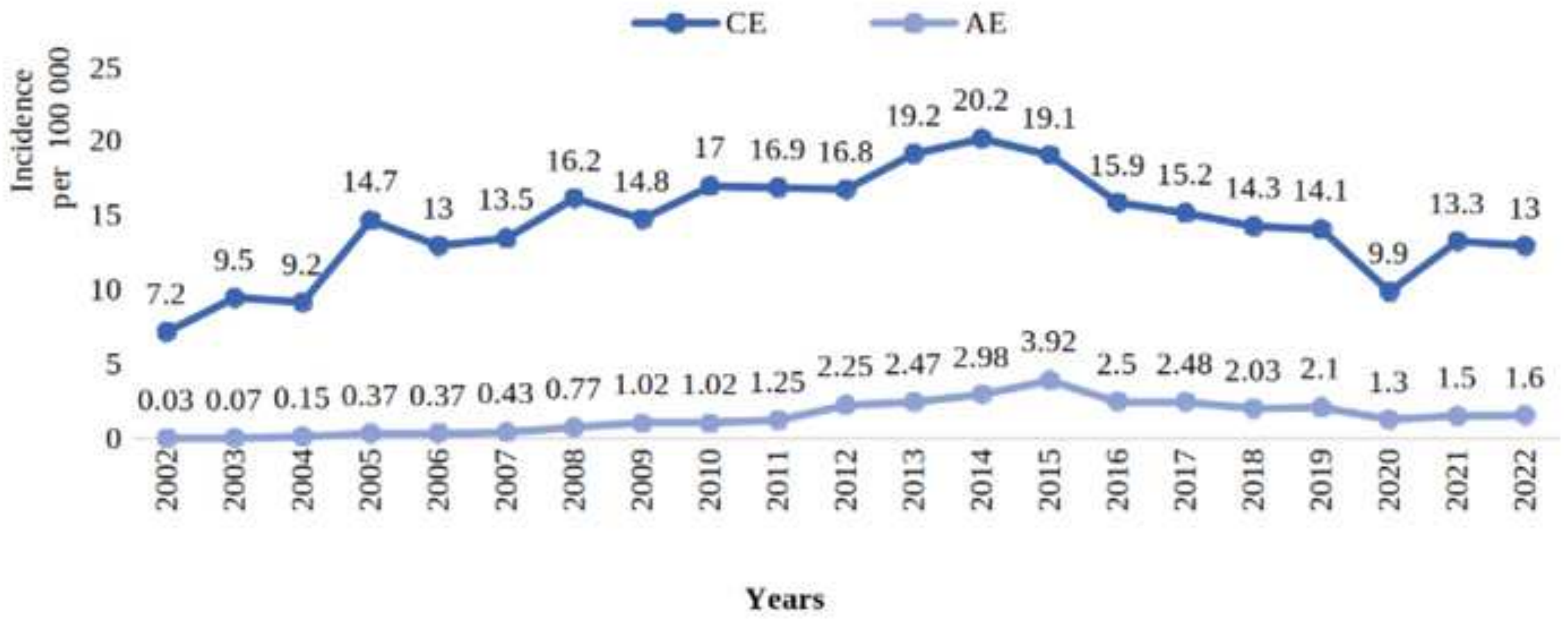

PLOS Neglected Tropical Diseases
ECONOMIC AND SOCIAL BURDEN OF CYSTIC AND ALVEOLAR ECHINOCOCCOSIS IN THE KYRGYZ REPUBLIC
PNTD-D-25-01461

Comments by the reviewer are in normal text. Our responses are in **bold.**

Reviewer 1.

Comment

Title
Title: Burden of diseases is mortality, morbidity and economic. Social aspect was not directly addressed. Therefore, I suggest deleting the words ' economic and social'

Response
Title changed as suggested OK

Comment

Introduction
Line 26: the measure of severity is case fatality rate (CFR). What is CFR for CE?
One study in 2017 reported 'average annual death rate from echinococcosis is 0.007 per 10,000 population, and the mortality is 1.29 (per 100 patients).". Is this considered sever? like rabies for instance with CFR=100%

Response
Text edited accordingly, including a citation to the reviewer‘s suggested CFR. OK

Comment
Line 35: Former Soviet Union. Mention modern day names.

Resposne
There are 15 new independent states of the former Soviet Union and it would be cumbersome to list them all. So we think it is better left as it is in this context.

Comment
Line 42: Better to say 'may cause sever disease...'

Response
text edited as suggested OK

Comment
Line 51: report prevalence per 100,000. The 15% and 4% prevalence may reflect the infection rate in the study sample! Check.

Response.

The major difference, IR includes new case while prevalence is ALL cases. Both can be reported per 100, 1000, etc. (Ref. Gordis Epidemiology). The question was about the national prevalence and not the infection rate of a given study sample.

It is not correct to report prevalence per 100,000. Prevalence is a simple proportion of individual affected by the disease at a single time point and is normally reported as a proportion or %. This is not to be confused with incidence, which is reported as number of cases per 100,000 per year. So reporting prevalence as cases per 100,000 would confuse the reader. Incidence and prevalence are different concepts in measuring disease occurrence and it is important they are not confused. Therefore we do not think it is correct to make changes here.

Comment
Line 54: Is it Kyrgyzstan or China? Check.

Response
It is both. They are separate examples. This is clear in the text and has separate bibliographic citations to China and Kyrgyzstan. So no change in the text is required **OK**

Comment
Is it possible to have a paragraph or two about burden of disease including costs for CE and AE?

Response
Now included in the introduction **OK**

Methods
Line 69: Is there a real document with number for the ethics approval.

Response
There is a document issued by the Committee on Bioethics (BEC) of the Kyrgyz State Medical Academy named after. I.K. Akhunbaeva. It is in Russian but has no number. **OK**

Comment
Line 79: Did it include the testing of CE and AE? and what type of diagnostic test is adopted for the case definition in Kyrgyzstan?

Response

Both AE and CE are notifiable diseases in Kyrgyzstan. This study was based on officially notified cases reported from hospitals treating patients. Diagnosis was based on imaging and confirmation by histopathology following surgical treatment of cases. No additional text necessary. **OK**

Comment
Line 85-89: can be rephrased to mention a five year age group from zero to 85 yr.
Line 94: Mention briefly the standard method and add the DALY equations used. Mention the reference life expectancy used in the calculations.

Response
Detailed methodology is referred to, so no need to add further details. Life table (ie expectancy) is already given (the WHO life table [15])! **OK**

Comment

Line 108: Is it Fisher's exact test? Add more details to show how the calculations were done such as including equations, software, significant levels, etc. Steps to follow, in case someone wants to repeat the study.

Response
Yes Fisher‘s exact test. R is now cited. Equations for fisher test etc are not necessary as it is widely used standard test. **Modify: Fisher exact test using SPSS (for example) with a level of significance of P<0.05 (or similar).**

Comment
Results
Line 12: Does the graph show surgical incidence?

Response
It shows the numbers of confirmed cases that were officially notified to the Ministry of Health. Explanatory text has been inserted. **OK**

Table 1: The calculations should be revisited. Why is 95% CI needed? This is just a simple percentage.

Response
CIs are required as it is a sample and are calculated as exact binomial CIs.. It also allows easy comparison between proportion of recurrent cases between years and hence if there is any trend. **OK**

Comment
Line 129: It is better to say six-year prevalence rather than 'dynamic"

Response
Prevalence is not reported. It is number of incidence cases. Text has been clarified to that effect. **OK**

Comment
Line 32: In the parenthesis along with the P-value, include odds ratio, CI. Mention the type of test used.

Response
Fisher test is used and is stated in materials and methods. Data is given so OR not necessary **OK**

Comment
Table 2 can be represented in this way to be clearer to the reader with explicit statistics (see suggested table below). Table 1 can be thought of in a similar way

	CE	AE	CFR	OR (95% CI)	P-Value	CE
2015	Mortality	?	?	=deaths/diseased x100	? (?-?)	??
	Morbidity	?	?			
2016	Mortality	?	?		? (?-?)	??
	Morbidity	?	?			
2017	Mortality	?	?		? (?-?)	??
	Morbidity	?	?			

Total	Mortality	55	36		0.27 (0.18-0.42)	0.0001
	Morbidity	6521	1187			

Response
This is not entirely clear what the reviewer wants. We have changed the table so it included numbers of cases, fatal cases and case fatality rate. OR is not applicable to this data. **The modifications to table 2 are enough**

Comment
Table 3: Check if it should be a comma or a period that separates the numbers in the table.

Response
Period is now used. **OK**

Comment
Add all abbreviations mentioned in the table in full beneath

Response
Corrected where appropriate **OK**

Comment
Line 157: What is KGS?

Response
KGS is Kyrgyz Som. I would refer the reviewer to the materials and methods where this abbreviation was already defined. **OK**

Comment
What is the percentage of expenses for the disease (CE and AE) out of the total medical expenses? Is it possible to make a breakdown of how this number was reached?

Response
This information is already given in the tables **OK**

Comment
Line 158: It would be a good idea to restrict the costs to USD.

Response
The study was undertaken in Kyrgyzstan. The local currancy is KGS. Therefore it is better to quote both figures **OK**

Comment
Line 159: was the 8580KGS the mean of the CE (8376) and AE (9680) costs?

Response
Yes, this is already explained in the text **OK**

Comment
Line 162: can you explain briefly what PPE is?

Purchasing power equivalent, now defined in materials and methods **OK**

Comment
Table 4: I suggest to show costs in USD.

Response
Costs are already shown in USD, but also in KGS and PPE $ OK

Comment
Column 1: should be 'site of infection' not 'Diagnosis cystic echinococcosis'.

Response
`Site of primary lesion` would be most appropriate and has been edited accordingly OK

Comment
Column 2: should be cost per case. Better in USD.

Response
Local currancy USD and PPE preferred, see above response. OK

Comment
Column 3 and 4: Exchange rate columns are not needed.

Response
See previous comment

Comment
Column 5: should be ' number of cases'

Response
Yes! Corrected OK

Comment
Column 6: What does 95% CI indicate?

Response
Column 6 not necessary, so deleted OK

Comment

Column 6: In USD. Is it the multiplication of column 2 by column 5?
Last column is unnecessary if all in USD.
Try to modify the table in a way to make easier and more informative.

Response
Table modified accordingly PK

Comment
Table 5: costs better be in USD.

Response
Already included in summary costs OK

Comment
The links are in Kyrgze language. International reader will not benefit from it.
I suggest deleting them.

Response
The links are in Russian, but provide the source of data (important for reproducibility). Web translators can give translation to English.
I see that you have deleted the links

Comment
Column 2: USD/ CE case
Column 3: USD, total (n=6578)
Column 4: USD/ AE case

Response
Not necessary OK

Comment
Line 187: change to ' For Ae, it should have...'

Response
Edited accordingly OK

Comment
Discussions
Line 194-202: discuss the burden of disease (DALY and costs) rather than what has been written.

Response
There is already extensive discussion on this aspect in a succeeding paragraph. OK

Comment
Line: 203-206: Add a citation.

Response
It is unclear why a citation is needed. Please can the reviewer elaborate
There was a substantial decrease in the number of reported cases and deaths due to CE and AE in 2020, which is associated with the outbreak of Coronavirus. CITE THIS SENTENCE

Comment
Line 207: higher case fatality rate then CE, this should be mentioned.

Response
It has been! OK

Comment
Line 224: sentence is incomplete.

Response
The sentence appears complete to me.
'The high number of disability-adjusted life years – DALYs deaths demonstrates not only the deadliness of these diseases.' Either remove 'not only' or add 'but also' followed by other confirmatory words

Comment
Line 231: Rephrase 'This is because..'

Response
No such phrase at line 231. Maybe at line 212...but there it makes sense **OK**

Comment
Line 236-241: This paragraph should be moved up in the discussion.

Response
This paragraphs outlines the strengths of the study, and is generally places towards the end together with the weaknesses. So I am not sure why the reviewer would like to see it moved.
This paragraph is discussing the continuous increase of IR for CE and AE in the last two decades. It came after the DALY paragraph. It can be better incorporated in the second paragraph of the discussion which discuss the fatality and mortality

Comment
The burden of disease in terms of costs is not well-discussed. DALY numbers is nor discussed.

Response
Some extra text has been added about the DALYs in terms of DALYs per case. The financial cost appear to us to be well discussed, although perhaps the reviewer might like to advise further **OK**

Comment
Line 243: the public health challenges paragraphs are not benefiting from the study results and are benefiting the discusses due to their general nature. General wording is just filling space.

Response
This comment does not make sense. Could the reviewer reword then it can be addressed. **Not the job of reviewer**

Comment
Line 258-265: unnecessary.

Response
Text deleted **OK**

Comment
Line 269: Rephrase the sentence 'The study may miss..'

Response
Such text was not at line 269 or elsewhere **OK**

Comment
Line 277-293: Discussion should be focused on the explanation of the results. Such paragraph does not hit the purpose.

Response
Text deleted **OK**

Comment
Conclusion: A one-page conclusion is considered long. Make it short. The discussion is long, general, does not tackle the results in detailed manner. It needs a more related set of references.

Response
Conclusion has been considerably shortened by deletion of lines 277-293 as in previous comment. Further text has been removed and/or edited accordingly. **OK**

Reviewer 2

Reviewer Comments
General Assessment
This manuscript estimates the epidemiological and economic burden of cystic (CE) and alveolar echinococcosis (AE) in Kyrgyzstan using national surveillance (AIS) and insurance (CMIF) data for 2015–2020, and derives DALYs and treatment costs.
The topic is important and policy-relevant, and the dataset appears substantial. However, there are significant methodological and reporting issues that must be addressed before the results can be considered reliable.
Major Comments
Several strong statements about regional trends (former Soviet Union/Eastern Europe; Central Asia; Tibet/western China; Europe/Switzerland) are presented as background but without clear, up-to-date sourcing in-text. Ensure that each quantitative claim (incidence, prevalence, "increasing trend") has a current citation and that the numbers align with the cited time windows.

Response
Text edited accordingly.

Comment
You use multiple AIS categories (CE, AE, and "relapse" for each). Please provide the **case definitions** applied by AIS for CE vs AE, and the **operational definition of relapse** (same anatomical site? within how many months/years? surgery-confirmed? imaging-confirmed?).Provide uncertainty estimates if possible.

Response
Both diseases were cases based on clinical, pathological and histological confirmation of the lesion. Relapsed cases were those with the same diagnosis of CE or AE at the same anatomical site six or more months after discharge following treatment. This is now included in the text

Comment
Clarify whether relapse cases were excluded from incidence denominators or double-counted across years.

Response
This is clearly indicated in the tables

Response
Incidences in figures are new cases, ie exclude relapsed. A note in the caption now indices this. For the DALY calculation and economic costs total cases are used as the data for follow up (YLDs see later comment), and costs of treatemnt for relapses should be included.

Comment
Specify whether you used an incidence-based or prevalence-based YLD approach, and describe how duration was derived for CE and AE (by clinical pathway or literature) beyond the reference.

Response
YLDs were incidence based, now stated in materials and methods. Duration was the length of the follow up. This was already stated in the materials and methods.

Comment
You present both a nominal exchange rate (e.g. 85.5 KGS per USD) and a PPP conversion factor (21.74 KGS per international dollar). This is confusing as written. Clarify your approach in the methods section: state which rate was used for each calculation, the base year, and the source.

Response
Text edited to improve clarity (also see 1st reviewer). Citation for PPP and exchange rate. Base year 2022.

Line-by-Line / Specific Comments

**Line(s)**	**Comment / Correction**
33	“senso lata” → *sensu lato*<br>**Corrected**
34	“E. granulosis” → *E. granulosus*<br>**Corrected**
36	“dramatically increased” — specify **since when**<br>**Since 1991, when the USSR was disolved**
36–39	Add a **reference** for the increase.<br>**There was already a reference, but additional ones have been added,**
53	“100.000” → “100,000” (**apply throughout**)<br>**Corrected**
76–77	Remove repeated phrase: “Ministry of Health of the Kyrgyz Republic for 2015–2020”.<br>**Corrected**
102–103	You introduce “CMIF” but do not use it later — either use consistently or remove.<br>**Corrected**
103	“som” → “Kyrgyzstani Som”.<br>**Corrected**
113	“figure 1” → **Figure 1** (capitalise).
Figure 1	Caption states 2001–2022, but figure shows 2002–2022 — correct discrepancy.
117	“Table 1.” → **Table 1** (remove full stop).<br>**Corrected**
118–120	Sentence beginning *“For CE, primary cases accounted…”* — rewrite for clarity and fix

Line(s)	Comment / Correction
	punctuation.<br>**Corrected**
122–124	Sentence beginning *“Compared to 2015 and 2020…”* — remove the first mention of 2020; redundant and confusing.<br>**Corrected**
126–127	“Table 1 The ratio…” → “Table 1. The ratio of patients with primary n = 6,448 and recurrent n = 1,351 CE and AE treated in hospitals (n = 7,799) and the number of fatal cases (n = 91), Kyrgyz Republic, 2015–2020.” (also apply consistent formatting throughout).<br>**Corrected**
129–132	“What dynamics?” — please rewrite for clarity and fix punctuation.<br>**Corrected**
135	Use *CE* consistently after first definition; do not revert to full term mid-section.<br>**Corrected. Also AE**
137	Correct punctuation around *p*-value (e.g. $p < 0.05$).<br>**Corrected**
141	“table 3” → **Table 3** (also throughout).<br>**Corrected**
143–144	Remove duplication: “The, the annual burden”.<br>**Corrected**
Table 3	Include definitions for abbreviations in caption (e.g. WHO GHE). Ensure “(AE)” appears in the correct section heading.<br>**Corrected**
153–154	If abbreviations (e.g. CMIF) are introduced, use them consistently thereafter.<br>**Corrected**
155	“7 799” → “7,799”.<br>**Corrected**
167	Spell out “three”.

Line(s)	Comment / Correction
	**Corrected**
169	“alveolar echinococcosis” → “AE”.<br>**Corrected**
168–170	Be consistent in formatting of “US $” or “USD” throughout.<br>**Corrected**
Table 4	The table reads poorly due to column-label formatting. Clarify all columns and headings, especially “Exchange rate 85.50”. Move exchange rates and PPP factor to caption. Place total counts first for readability. Explain the meaning of the 95% CI column — why does it have both a value and a range? Reformat and relabel for clarity.<br>**Corrected and clarified. Also removed the ranges**
Table 5	Provide references for cost values in the **Methods**, not within the table.<br>**Corrected**
186–188	Replace conditional “should have amounted” with “has amounted”. Also change “10,5 million” → “10.5 million”.<br>**Corrected**
190	“dollarsout” → “dollars out”.<br>**Corrected**
208–209	Clarify “low according/in relation to what?” — add comparison or context.<br>**We are uncertain as to which text this refers to. Can the referee clarify**
231	“Coulotte et al. [14]” → “Coulotte et al. [14].” (add final period).<br>**Corrected**
236–239	Corrected sentence:<br>**“The annual incidence of CE in Kyrgyzstan, based on hospital records, increased from 5.4 cases/100,000 in 1991 to 14 cases/100,000 in 1998 [5], and then to 20 cases/100,000 in 2014 (the present study). Between 1995 and 2011, human AE increased from < 3 cases per year to > 60 cases per year, and then to over 200 per year by 2015 [11, 18].”**<br>**Corrected**
243	Ensure all **subtitles** are correctly formatted (consistent style and hierarchy).
258–265	The **“Strengths of the study”** paragraph is unnecessary; consider removing.<br>**OK removed**

Line(s)	Comment / Correction
	Sample sizes vary between 7,789 and 7,799 in different sections — reconcile all totals.
—	**Corrected, it is 7,799**

Click here to access/download
Revised Article with Changes Highlighted
MGA_revised.docx
