# Supplementary material for: Burden of cystic and alveolar echinococcosis in the Kyrgyz Republic
Source: PLoS Negl Trop Dis. 2026 Mar 23;20(3):e0014074. doi: 10.1371/journal.pntd.0014074 (PMC13038107; doi:10.1371/journal.pntd.0014074)
Supplement: Attachment — Submitted filename: PNTD-D-25-01461_reviewer.doc [file pntd.0014074.s001.doc]

PLOS Neglected Tropical Diseases
ECONOMIC AND SOCIAL BURDEN OF CYSTIC AND ALVEOLAR
ECHINOCOCCOSIS IN THE KYRGYZ REPUBLIC

PNTD-D-25-01461

**Title**

Title: Burden of diseases is mortality, morbidity and economic. Social aspect was not directly addressed. Therefore, I suggest deleting the words ' economic and social'

**Introduction**

Line 26: the measure of severity is case fatality rate (CFR). What is CFR for CE?

One study in 2017 reported 'average annual death rate from echinococcosis is 0.007 per 10,000 population, and the mortality is 1.29 (per 100 patients).". Is this considered sever? like rabies for instance with CFR=100%

Line 35: Former Soviet Union. Mention modern day names.

Line 42: Better to say 'may cause sever disease...'

Line 51: report prevalence per 100,000. The 15% and 4% prevalence may reflect the infection rate in the study sample! Check.

Line 54: Is it Kyrgyzstan or China? Check.

Is it possible to have a paragraph or two about burden of disease including costs for CE and AE?

**Methods**

Line 69: Is there a real document with number for the ethics approval.

Line 79: Did it include the testing of CE and AE? and what type of diagnostic test is adopted for the case definition in Kyrgyzstan?

Line 85-89: can be rephrased to mention a five year age group from zero to 85 yr.

Line 94: Mention briefly the standard method and add the DALY equations used. Mention the reference life expectancy used in the calculations.

Line 108: Is it Fisher's exact test? Add more details to show how the calculations were done such as including equations, software, significant levels, etc. Steps to follow, in case someone wants to repeat the study.

**Results**

Line 12: Does the graph show surgical incidence?

Table 1: The calculations should be revisited. Why is 95% CI needed? This is just a simple percentage.

Line 129: It is better to say six-year prevalence rather than 'dynamic"

Line 32: In the parenthesis along with the P-value, include odds ratio, CI. Mention the type of test used.

Table 2 can be represented in this way to be clearer to the reader with explicit statistics (see suggested table below). Table 1 can be thought of in a similar way

|  | CE | AE | CFR | OR (95% CI) | P-Value | CE |
| --- | --- | --- | --- | --- | --- | --- |
| 2015 | Mortality | ? | ? | =deaths/diseased x100 | ? (?-?) | ?? |
|  | Morbidity | ? | ? |  |  |  |
| 2016 | Mortality | ? | ? |  | ? (?-?) | ?? |
|  | Morbidity | ? | ? |  |  |  |
| 2017 | Mortality | ? | ? |  | ? (?-?) | ?? |
|  | Morbidity | ? | ? |  |  |  |
|  |  |  |  |  |  |  |
|  |  |  |  |  |  |  |
|  |  |  |  |  |  |  |
|  |  |  |  |  |  |  |
| Total | Mortality | 55 | 36 |  | 0.27 (0.18-0.42) | 0.0001 |
|  | Morbidity | 6521 | 1187 |  |  |  |

Table 3: Check if it should be a comma or a period that separates the numbers in the table.

Add all abbreviations mentioned in the table in full beneath

Line 157: What is KGS? What is the percentage of expenses for the disease (CE and AE) out of the total medical expenses? Is it possible to make a breakdown of how this number was reached?

Line 158: It would be a good idea to restrict the costs to USD.

Line 159: was the 8580KGS the mean of the CE (8376) and AE (9680) costs?

Line 162: can you explain briefly what PPE is?

Table 4: I suggest to show costs in USD.

Column 1: should be 'site of infection' not 'Diagnosis cystic chinococcosis'.

Column 2: should be cost per case. Better in USD.

Column 3 and 4: Exchange rate columns are not needed.

Column 5: should be ' number of cases'

Column 6: What does 95% CI indicate?

Column 6: In USD. Is it the multiplication of column 2 by column 5?

Last column is unnecessary if all in USD.

Try to modify the table in a way to make easier and more informative.

Table 5: costs better be in USD.

The links are in Kyrgze language. International reader will not benefit from it. I suggest deleting them.

Column 2: USD/ CE case

Column 3: USD, total (n=6578)

Column 4: USD/ AE case

Line 187: change to ' For Ae, it should have...'

**Discussions**

Line 194-202: discuss the burden of disease (DALY and costs) rather than what has been written.

Line: 203-206: Add a citation.

Line 207: higher case fatality rate then CE, this should be mentioned.

Line 224: sentence is incomplete.

Line 231: Rephrase 'This is because..'

Line 236-241: This paragraph should be moved up in the discussion.

The burden of disease in terms of costs is not well-discussed. DALY numbers is nor discussed.

Line 243: the public health challenges paragraphs are not benefiting from the study results and are benefiting the discusses due to their general nature. General wording is just filling space.

Line 258-265: unnecessary.

Line 269: Rephrase the sentence 'The study may miss..'

Line 277-293: Discussion should be focused on the explanation of the results. Such paragraph does not hit the purpose.

Conclusion: A one-page conclusion is considered long. Make it short. The discussion is long, general, does not tackle the results in detailed manner. It needs a more related set of references.
