# Supplementary material for: Burden of cystic and alveolar echinococcosis in the Kyrgyz Republic
Source: PLoS Negl Trop Dis. 2026 Mar 23;20(3):e0014074. doi: 10.1371/journal.pntd.0014074 (PMC13038107; doi:10.1371/journal.pntd.0014074)
Supplement: Attachment — Submitted filename: Review.docx [file pntd.0014074.s002.docx]

**Reviewer Comments**

**General Assessment**

This manuscript estimates the epidemiological and economic burden of cystic (CE) and alveolar echinococcosis (AE) in Kyrgyzstan using national surveillance (AIS) and insurance (CMIF) data for 2015–2020, and derives DALYs and treatment costs.
The topic is important and policy-relevant, and the dataset appears substantial. However, there are significant methodological and reporting issues that must be addressed before the results can be considered reliable.

**Major Comments**
Several strong statements about regional trends (former Soviet Union/Eastern Europe; Central Asia; Tibet/western China; Europe/Switzerland) are presented as background but without clear, up-to-date sourcing in-text. Ensure that each quantitative claim (incidence, prevalence, “increasing trend”) has a current citation and that the numbers align with the cited time windows.
You use multiple AIS categories (CE, AE, and “relapse” for each). Please provide the **case definitions** applied by AIS for CE vs AE, and the **operational definition of relapse** (same anatomical site? within how many months/years? surgery-confirmed? imaging-confirmed?).
Clarify whether relapse cases were excluded from incidence denominators or double-counted across years.
Specify whether you used an incidence-based or prevalence-based YLD approach, and describe how duration was derived for CE and AE (by clinical pathway or literature) beyond the reference. Provide uncertainty estimates if possible.
You present both a nominal exchange rate (e.g. 85.5 KGS per USD) and a PPP conversion factor (21.74 KGS per international dollar). This is confusing as written. Clarify your approach in the methods section: state which rate was used for each calculation, the base year, and the source.

**Line-by-Line / Specific Comments**

| **Line(s)** | **Comment / Correction** |
| --- | --- |
| 33 | “senso lata” → *sensu lato* |
| 34 | “E. granulosis” → *E. granulosus* |
| 36 | “dramatically increased” — specify **since when** |
| 36–39 | Add a **reference** for the increase. |
| 53 | “100.000” → “100,000” (**apply throughout**) |
| 76–77 | Remove repeated phrase: “Ministry of Health of the Kyrgyz Republic for 2015–2020”. |
| 102–103 | You introduce “CMIF” but do not use it later — either use consistently or remove. |
| 103 | “som” → “Kyrgyzstani Som”. |
| 113 | “figure 1” → **Figure 1** (capitalise). |
| Figure 1 | Caption states 2001–2022, but figure shows 2002–2022 — correct discrepancy. |
| 117 | “Table 1.” → **Table 1** (remove full stop). |
| 118–120 | Sentence beginning *“For CE, primary cases accounted…”* — rewrite for clarity and fix punctuation. |
| 122–124 | Sentence beginning *“Compared to 2015 and 2020…”* — remove the first mention of 2020; redundant and confusing. |
| 126–127 | “Table 1 The ratio…” → **“Table 1. The ratio of patients with primary n = 6,448 and recurrent n = 1,351 CE and AE treated in hospitals (n = 7,799) and the number of fatal cases (n = 91), Kyrgyz Republic, 2015–2020.”** (also apply consistent formatting throughout). |
| 129–132 | “What dynamics?” — please rewrite for clarity and fix punctuation. |
| 135 | Use *CE* consistently after first definition; do not revert to full term mid-section. |
| 137 | Correct punctuation around *p*-value (e.g. *p* < 0.05). |
| 141 | “table 3” → **Table 3** (also throughout). |
| 143–144 | Remove duplication: “The, the annual burden”. |
| Table 3 | Include definitions for abbreviations in caption (e.g. WHO GHE). Ensure “(AE)” appears in the correct section heading. |
| 153–154 | If abbreviations (e.g. CMIF) are introduced, use them consistently thereafter. |
| 155 | “7 799” → “7,799”. |
| 167 | Spell out “three”. |
| 169 | “alveolar echinococcosis” → “AE”. |
| 168–170 | Be consistent in formatting of “US $” or “USD” throughout. |
| Table 4 | The table reads poorly due to column-label formatting. Clarify all columns and headings, especially “Exchange rate 85.50”. Move exchange rates and PPP factor to caption. Place total counts first for readability. Explain the meaning of the 95% CI column — why does it have both a value and a range? Reformat and relabel for clarity. |
| Table 5 | Provide references for cost values in the **Methods**, not within the table. |
| 186–188 | Replace conditional “should have amounted” with “has amounted”. Also change “10,5 million” → “10.5 million”. |
| 190 | “dollarsout” → “dollars out”. |
| 208–209 | Clarify “low according/in relation to what?” — add comparison or context. |
| 231 | “Coulotte et al. [14]” → “Coulotte et al. [14].” (add final period). |
| 236–239 | Corrected sentence: **“The annual incidence of CE in Kyrgyzstan, based on hospital records, increased from 5.4 cases/100,000 in 1991 to 14 cases/100,000 in 1998 [5], and then to 20 cases/100,000 in 2014 (the present study). Between 1995 and 2011, human AE increased from < 3 cases per year to > 60 cases per year, and then to over 200 per year by 2015 [11, 18].”** |
| 243 | Ensure all **subtitles** are correctly formatted (consistent style and hierarchy). |
| 258–265 | The **“Strengths of the study”** paragraph is unnecessary; consider removing. |
| — | Sample sizes vary between 7,789 and 7,799 in different sections — reconcile all totals. |
