# Supplementary material for: Burden of cystic and alveolar echinococcosis in the Kyrgyz Republic
Source: PLoS Negl Trop Dis. 2026 Mar 23;20(3):e0014074. doi: 10.1371/journal.pntd.0014074 (PMC13038107; doi:10.1371/journal.pntd.0014074)
Supplement: Attachment — Submitted filename: PNTD-D-25-01461_reviewer-response.doc [file pntd.0014074.s003.doc]

PLOS Neglected Tropical Diseases
ECONOMIC AND SOCIAL BURDEN OF CYSTIC AND ALVEOLAR
ECHINOCOCCOSIS IN THE KYRGYZ REPUBLIC

PNTD-D-25-01461

Comments by the reviewer are in normal text. Our responses are in **bold.**

Reviewer 1.

Comment

Title

Title: Burden of diseases is mortality, morbidity and economic. Social aspect was not directly addressed. Therefore, I suggest deleting the words ' economic and social'

**Response**

**Title changed as suggested**

Comment

Introduction

Line 26: the measure of severity is case fatality rate (CFR). What is CFR for CE?

One study in 2017 reported 'average annual death rate from echinococcosis is 0.007 per 10,000 population, and the mortality is 1.29 (per 100 patients).". Is this considered sever? like rabies for instance with CFR=100%

**Response**

**Text edited accordingly, including a citation to the reviewer‘s suggested CFR.**

Comment

Line 35: Former Soviet Union. Mention modern day names.

**Resposne**

**There are 15 new independent states of the former Soviet Union and it would be cumbersome to list them all. So we think it is better left as it is in this context.**

Comment

Line 42: Better to say 'may cause sever disease...'

**Response**

**text edited as suggested**

Comment

Line 51: report prevalence per 100,000. The 15% and 4% prevalence may reflect the infection rate in the study sample! Check.

**Response.**

**It is not correct to report prevalence per 100,000. Prevalence is a simple proportion of individual affected by the disease at a single time point and is normally reported as a proportion or %. This is not to be confused with incidence, which is reported as number of cases per 100,000 per year. So reporting prevalence as cases per 100,000 would confuse the reader. Incidence and prevalence are different concepts in measuring disease occurrence and it is important they are not confused. Therefore we do not think it is correct to make changes here.**

Comment

Line 54: Is it Kyrgyzstan or China? Check.

**Response**

**It is both. They are separate examples. This is clear in the text and has separate bibliographic citations to China and Kyrgyzstan. So no change in the text is required**

Comment

Is it possible to have a paragraph or two about burden of disease including costs for CE and AE?

**Response**

**Now included in the introduction**

**Methods**

Line 69: Is there a real document with number for the ethics approval.

**Response**

**There is a document issued by the Committee on Bioethics (BEC) of the Kyrgyz State Medical Academy named after. I.K. Akhunbaeva. It is in Russian but has no number.**

Comment

Line 79: Did it include the testing of CE and AE? and what type of diagnostic test is adopted for the case definition in Kyrgyzstan?

**Response**

**Both AE and CE are notifiable diseases in Kyrgyzstan. This study was based on officially notified cases reported from hospitals treating patients. Diagnosis was based on imaging and confirmation by histopathology following surgical treatment of cases. No additional text necessary.**

Comment

Line 85-89: can be rephrased to mention a five year age group from zero to 85 yr.

Line 94: Mention briefly the standard method and add the DALY equations used. Mention the reference life expectancy used in the calculations.

**Response**

**Detailed methodology is referred to, so no need to add further details. Life table (ie expectancy) is already given (the WHO life table [15])!**

Comment

Line 108: Is it Fisher's exact test? Add more details to show how the calculations were done such as including equations, software, significant levels, etc. Steps to follow, in case someone wants to repeat the study.

**Response**

**Yes Fisher‘s exact test. R is now cited. Equations for fisher test etc are not necessary as it is widely used standard test.**

**Comment**

**Results**

Line 12: Does the graph show surgical incidence?

**Response**

**It shows the numbers of confirmed cases that were officially notified to the Ministry of Health. Explanatory text has been inserted.**

Table 1: The calculations should be revisited. Why is 95% CI needed? This is just a simple percentage.

**Response**

**CIs are required as it is a sample and are calculated as exact binomial CIs.. It also allows easy comparison between proportion of recurrent cases between years and hence if there is any trend.**

Comment

Line 129: It is better to say six-year prevalence rather than 'dynamic"

**Response**

**Prevalence is not reported. It is number of incidence cases. Text has been clarified to that effect.**

Comment

Line 32: In the parenthesis along with the P-value, include odds ratio, CI. Mention the type of test used.

**Response**

**Fisher test is used and is stated in materials and methods. Data is given so OR not necessary**

Comment

Table 2 can be represented in this way to be clearer to the reader with explicit statistics (see suggested table below). Table 1 can be thought of in a similar way

|  | CE | AE | CFR | OR (95% CI) | P-Value | CE |
| --- | --- | --- | --- | --- | --- | --- |
| 2015 | Mortality | ? | ? | =deaths/diseased x100 | ? (?-?) | ?? |
|  | Morbidity | ? | ? |  |  |  |
| 2016 | Mortality | ? | ? |  | ? (?-?) | ?? |
|  | Morbidity | ? | ? |  |  |  |
| 2017 | Mortality | ? | ? |  | ? (?-?) | ?? |
|  | Morbidity | ? | ? |  |  |  |
|  |  |  |  |  |  |  |
|  |  |  |  |  |  |  |
|  |  |  |  |  |  |  |
|  |  |  |  |  |  |  |
| Total | Mortality | 55 | 36 |  | 0.27 (0.18-0.42) | 0.0001 |
|  | Morbidity | 6521 | 1187 |  |  |  |

**Response**

**This is not entirely clear what the reviewer wants. We have changed the table so it included numbers of cases, fatal cases and case fatality rate. OR is not applicable to this data.**

Comment

Table 3: Check if it should be a comma or a period that separates the numbers in the table.

**Response**

**Period is now used.**

Comment

Add all abbreviations mentioned in the table in full beneath

**Response**

**Corrected where appropriate**

Comment

Line 157: What is KGS?

**Response**

**KGS is Kyrgyz Som. I would refer the reviewer to the materials and methods where this abbreviation was already defined.**

Comment

What is the percentage of expenses for the disease (CE and AE) out of the total medical expenses? Is it possible to make a breakdown of how this number was reached?

**Response**

**This information is already given in the tables**

Comment

Line 158: It would be a good idea to restrict the costs to USD.

**Response**

**The study was undertaken in Kyrgyzstan. The local currancy is KGS. Therefore it is better to quote both figures**

Comment

Line 159: was the 8580KGS the mean of the CE (8376) and AE (9680) costs?

**Response**

**Yes, this is already explained in the text**

Comment

Line 162: can you explain briefly what PPE is?

**Purchasing power equivalent, now defined in materials and methods**

Comment

Table 4: I suggest to show costs in USD.

**Response**

**Costs are already shown in USD, but also in KGS and PPE $**

Comment

Column 1: should be 'site of infection' not 'Diagnosis cystic echinococcosis'.

**Response**

**`Site of primary lesion` would be most appropriate and has been edited accordingly**

Comment

Column 2: should be cost per case. Better in USD.

**Response**

**Local currancy USD and PPE preferred, see above response.**

Comment

Column 3 and 4: Exchange rate columns are not needed.

**Response**

**See previous comment**

Comment

Column 5: should be ' number of cases'

**Response**

**Yes! Corrected**

Comment

Column 6: What does 95% CI indicate?

**Response**

**Column 6 not necessary, so deleted**

Comment

Column 6: In USD. Is it the multiplication of column 2 by column 5?

Last column is unnecessary if all in USD.

Try to modify the table in a way to make easier and more informative.

**Response**

**Table modified accordingly**

Comment

Table 5: costs better be in USD.

**Response**

**Already included in summary costs**

Comment

The links are in Kyrgze language. International reader will not benefit from it.

I suggest deleting them.

**Response**

**The links are in Russian, but provide the source of data (important for reproducibility). Web translators can give translation to English.**

Comment

Column 2: USD/ CE case

Column 3: USD, total (n=6578)

Column 4: USD/ AE case

**Response**

**Not necessary**

Comment

Line 187: change to ' For Ae, it should have...'

**Response**

**Edited accordingly**

Comment

Discussions

Line 194-202: discuss the burden of disease (DALY and costs) rather than what has been written.

**Response**

**There is already extensive discussion on this aspect in a succeeding paragraph.**

Comment

Line: 203-206: Add a citation.

**Response**

**It is unclear why a citation is needed. Please can the reviewer elaborate**

Comment

Line 207: higher case fatality rate then CE, this should be mentioned.

**Response**

**It has been!**

Comment

Line 224: sentence is incomplete.

**Response**

**The sentence appears complete to me.**

Comment

Line 231: Rephrase 'This is because..'

**Response**

**No such phrase at line 231. Maybe at line 212...but there it makes sense**

Comment

Line 236-241: This paragraph should be moved up in the discussion.

**Response**

**This paragraphs outlines the strengths of the study, and is generally places towards the end together with the weaknesses. So I am not sure why the reviewer would like to see it moved.**

Comment

The burden of disease in terms of costs is not well-discussed. DALY numbers is nor discussed.

**Response**

**Some extra text has been added about the DALYs in terms of DALYs per case. The financial cost appear to us to be well discussed, although perhaps the reviewer might like to advise further**

Comment

Line 243: the public health challenges paragraphs are not benefiting from the study results and are benefiting the discusses due to their general nature. General wording is just filling space.

**Response**

**This comment does not make sense. Could the reviewer reword then it can be addressed.**

Comment

Line 258-265: unnecessary.

**Response**

**Text deleted**

Comment

Line 269: Rephrase the sentence 'The study may miss..'

**Response**

**Such text was not at line 269 or elsewhere**

Comment

Line 277-293: Discussion should be focused on the explanation of the results. Such paragraph does not hit the purpose.

**Response**

**Text deleted**

Comment

Conclusion: A one-page conclusion is considered long. Make it short. The discussion is long, general, does not tackle the results in detailed manner. It needs a more related set of references.

**Response**

**Conclusion has been considerably shortened by deletion of lines 277-293 as in previous comment. Further text has been removed and/or edited accordingly.**

**Reviewer 2**

**Reviewer Comments**

**General Assessment**

This manuscript estimates the epidemiological and economic burden of cystic (CE) and alveolar echinococcosis (AE) in Kyrgyzstan using national surveillance (AIS) and insurance (CMIF) data for 2015–2020, and derives DALYs and treatment costs.
The topic is important and policy-relevant, and the dataset appears substantial. However, there are significant methodological and reporting issues that must be addressed before the results can be considered reliable.

**Major Comments**
Several strong statements about regional trends (former Soviet Union/Eastern Europe; Central Asia; Tibet/western China; Europe/Switzerland) are presented as background but without clear, up-to-date sourcing in-text. Ensure that each quantitative claim (incidence, prevalence, “increasing trend”) has a current citation and that the numbers align with the cited time windows.

**Response**

**Text edited accordingly.**

Comment
You use multiple AIS categories (CE, AE, and “relapse” for each). Please provide the **case definitions** applied by AIS for CE vs AE, and the **operational definition of relapse** (same anatomical site? within how many months/years? surgery-confirmed? imaging-confirmed?).Provide uncertainty estimates if possible.

**Response**

**Both diseases were cases based on clinical, pathological and histological confirmation of the lesion. Relapsed cases were those with the same diagnosis of CE or AE at the same anatomical site six or more months after discharge following treatment. This is now included in the text**

Comment

Clarify whether relapse cases were excluded from incidence denominators or double-counted across years.

**Response**

**This is clearly indicated in the tables**

**Response**

**Incidences in figures are new cases, ie exclude relapsed. A note in the caption now indices this. For the DALY calculation and economic costs total cases are used as the data for follow up (YLDs see later comment), and costs of treatemnt for relapses should be included.**

Comment

Specify whether you used an incidence-based or prevalence-based YLD approach, and describe how duration was derived for CE and AE (by clinical pathway or literature) beyond the reference.

**Response**

**YLDs were incidence based, now stated in materials and methods. Duration was the length of the follow up. This was already stated in the materials and methods.**

Comment

You present both a nominal exchange rate (e.g. 85.5 KGS per USD) and a PPP conversion factor (21.74 KGS per international dollar). This is confusing as written. Clarify your approach in the methods section: state which rate was used for each calculation, the base year, and the source.

**Response**

**Text edited to improve clarity (also see 1^st^ reviewer). Citation for PPP and exchange rate. Base year 2022.**

**Line-by-Line / Specific Comments**

| **Line(s)** | **Comment / Correction** |
| --- | --- |
| 33 | “senso lata” → *sensu lato*  **Corrected** |
| 34 | “E. granulosis” → *E. granulosus*  **Corrected** |
| 36 | “dramatically increased” — specify **since when**  **Since 1991, when the USSR was disolved** |
| 36–39 | Add a **reference** for the increase.  **There was already a reference, but additional ones have been added,** |
| 53 | “100.000” → “100,000” (**apply throughout**)  **Corrected** |
| 76–77 | Remove repeated phrase: “Ministry of Health of the Kyrgyz Republic for 2015–2020”.  **Corrected** |
| 102–103 | You introduce “CMIF” but do not use it later — either use consistently or remove.  **Corrected** |
| 103 | “som” → “Kyrgyzstani Som”.  **Corrected** |
| 113 | “figure 1” → **Figure 1** (capitalise). |
| Figure 1 | Caption states 2001–2022, but figure shows 2002–2022 — correct discrepancy. |
| 117 | “Table 1.” → **Table 1** (remove full stop).  **Corrected** |
| 118–120 | Sentence beginning *“For CE, primary cases accounted…”* — rewrite for clarity and fix punctuation.  **Corrected** |
| 122–124 | Sentence beginning *“Compared to 2015 and 2020…”* — remove the first mention of 2020; redundant and confusing.  **Corrected** |
| 126–127 | “Table 1 The ratio…” → “Table 1. The ratio of patients with primary n = 6,448 and recurrent n = 1,351 CE and AE treated in hospitals (n = 7,799) and the number of fatal cases (n = 91), Kyrgyz Republic, 2015–2020.” (also apply consistent formatting throughout).  **Corrected** |
| 129–132 | “What dynamics?” — please rewrite for clarity and fix punctuation.  **Corrected** |
| 135 | Use *CE* consistently after first definition; do not revert to full term mid-section.  **Corrected. Also AE** |
| 137 | Correct punctuation around *p*-value (e.g. *p* < 0.05).  **Corrected** |
| 141 | “table 3” → **Table 3** (also throughout).  **Corrected** |
| 143–144 | Remove duplication: “The, the annual burden”.  **Corrected** |
| Table 3 | Include definitions for abbreviations in caption (e.g. WHO GHE). Ensure “(AE)” appears in the correct section heading.  **Corrected** |
| 153–154 | If abbreviations (e.g. CMIF) are introduced, use them consistently thereafter.  **Corrected** |
| 155 | “7 799” → “7,799”.  **Corrected** |
| 167 | Spell out “three”.  **Corrected** |
| 169 | “alveolar echinococcosis” → “AE”.  **Corrected** |
| 168–170 | Be consistent in formatting of “US $” or “USD” throughout.  **Corrected** |
| Table 4 | The table reads poorly due to column-label formatting. Clarify all columns and headings, especially “Exchange rate 85.50”. Move exchange rates and PPP factor to caption. Place total counts first for readability. Explain the meaning of the 95% CI column — why does it have both a value and a range? Reformat and relabel for clarity.  **Corrected and clarified. Also removed the ranges** |
| Table 5 | Provide references for cost values in the **Methods**, not within the table.  **Corrected** |
| 186–188 | Replace conditional “should have amounted” with “has amounted”. Also change “10,5 million” → “10.5 million”.  **Corrected** |
| 190 | “dollarsout” → “dollars out”.  **Corrected** |
| 208–209 | Clarify “low according/in relation to what?” — add comparison or context.  **We are uncertain as to which text this refers to. Can the referee clarify** |
| 231 | “Coulotte et al. [14]” → “Coulotte et al. [14].” (add final period).  **Corrected** |
| 236–239 | Corrected sentence: **“The annual incidence of CE in Kyrgyzstan, based on hospital records, increased from 5.4 cases/100,000 in 1991 to 14 cases/100,000 in 1998 [5], and then to 20 cases/100,000 in 2014 (the present study). Between 1995 and 2011, human AE increased from < 3 cases per year to > 60 cases per year, and then to over 200 per year by 2015 [11, 18].”**  **Corrected** |
| 243 | Ensure all **subtitles** are correctly formatted (consistent style and hierarchy). |
| 258–265 | The **“Strengths of the study”** paragraph is unnecessary; consider removing.  **OK removed** |
| — | Sample sizes vary between 7,789 and 7,799 in different sections — reconcile all totals.  **Corrected, it is 7,799** |
