# Supplementary material for: Burden of cystic and alveolar echinococcosis in the Kyrgyz Republic
Source: PLoS Negl Trop Dis. 2026 Mar 23;20(3):e0014074. doi: 10.1371/journal.pntd.0014074 (PMC13038107; doi:10.1371/journal.pntd.0014074)
Supplement: Attachment — Submitted filename: BURDEN OF CYSTIC AND AL.docx [file pntd.0014074.s005.docx]

While the authors have addressed several methodological queries raised in the first round of reviews, such as defining recurrence and clarifying the DALY approach, the revised manuscript is currently not in a publishable state due to lapses in presentation, proofreading, and data formatting.

There is some discrepancy between the "Response to Reviewers" document and the actual manuscript text. The authors have stated that certain corrections were made, yet the manuscript text provided still contains the original phrasing. Tables 4 and 5 contain severe formatting errors and typos. The manuscript requires a thorough copy-edit and a meticulous check to ensure the English presented in the text in approriate.

Comments:

- Discrepancy between response letter and the manuscript in the response to Reviewer 2, regarding the comment on lines 186–188, the authors stated: "Corrected, replaced 'should have amounted' with 'has amounted'". However, the revised manuscript text still reads: "The total economic damage in terms of purchasing power equivalent (PPE) from CE should have amounted to more than 10,5 million... For AE should have amounted to more than 5.1 million...".
- Presentation of Table 4 is currently unacceptable for publication in its current form. The headers contain unprofessional errors such as "Internationao", "Internationaol", and "doller". The columns appear misaligned, making it difficult to verify the "Total Costs" against the "Cost per case".
- In the abstract, the authors state: "The proportion of fatal cases of AE was 3.6 times higher than from CE". However, later in the text, they state: "mortality in AE was 3.6 times higher than for CE". Please ensure consistent terminology. In epidemiology, "Mortality" typically refers to deaths per population denominator, whereas "Case Fatality Rate" refers to deaths per infected case denominator. Given the context, it appears the authors mean CFR, but precise terminology is required to avoid misleading the reader.

Corrections

Abstract:

"For AE was there were 3809 DALYs...".

"The purpose of this study is to assess..." – Please change to "was to assess" to maintain past tense consistency with the results.

"From 2015-2020 a total of..." -> "From 2015–2020, a total...".

Introduction:

"Echinococcosis is a highly pathogenic parasitic diseases...". disease instead of diseases

"CE not may cause severe disease..." -> “CE not only cause severe disease...”?.

Results:

Table 1: The column "Proportion Recurrent (95% CI)" uses commas for decimals in the total row (15.3% (13,7% to 16,8%)) but dots elsewhere (2.9%). Please standardise.

Discussion:

"There was a substantial decrease in the number of reported cases... in 2020, which is associated with the outbreak of Coronavirus.". Please rephrase "associated with" to something more precise if no causal analysis was done, such as "coincided with," or provide a citation linking the pandemic to reduced surveillance in Kyrgyzstan specifically.

References:

Several URLs in the reference list are raw links. These should be formatted with "Accessed on [Date]" and a descriptive title of the webpage in accordance to appropriate referencing standards.
