# Supplementary material for: Burden of cystic and alveolar echinococcosis in the Kyrgyz Republic
Source: PLoS Negl Trop Dis. 2026 Mar 23;20(3):e0014074. doi: 10.1371/journal.pntd.0014074 (PMC13038107; doi:10.1371/journal.pntd.0014074)
Supplement: Attachment — Submitted filename: Response_to_reveiwers round2.docx [file pntd.0014074.s006.docx]

**Response to reviewers, round 2.**

Below are the reviewers and editors comments comments and how we have addressed them.

**Editor**

Author summary has now been provided.

Ethics. Additional text has been added to the ethics statement...

This study was based on secondary data obtained from the “Automated Information System for Surveillance of Infectious and Parasitic Diseases” (AIS) of the Department of Disease Prevention and State Sanitary and Epidemiological Surveillance of the Ministry of Health of the Kyrgyz Republic for the period 2015–2020.

All data were provided in a fully anonymized and aggregated form. The database contained no personal identifiers (including names, addresses, contact information, or individual identification numbers), making it impossible to identify individual patients or to link records to specific persons.

All analyses and calculations were performed using aggregated anonymized data and were conducted in accordance with internationally accepted principles of bioethics. The analyses did not involve individual patient-level data and did not affect or target specific patients, thereby fully preserving confidentiality and anonymity.

**Reveiwer’s concerns**

Reviewer 1.

No comments to address

Reviewer 2.

Comments were made an attached file and are addressed as below.

Comment

The major difference, IR includes new cases while prevalence is ALL cases. Both can be reported per 100, 1000, etc (Ref. Gordis Epidemiology). The question was about the national prevalence and not the infection rate of a given study sample.

Response

Here we reiterate that the standard way of reporting incidence is new cases per 100,000 per year. IR is new cases across a time period, not merely “including” new cases. The question was not about national prevalence but merely citing local studies that reported the proportion of the population infected from the total numbers examined. Studies like that are usually cross sectional studies using ultrasound to detect abdominal cysts and hence are point estimates in a small defined population. Hence these were local prevalences, not national prevalences and were cited to illustrate the possible extent of echinococcosis beyond official reported cases. National prevalence is unknown and can only be estimated by a suitable cross sectional study across a representative population samples. Yes sometimes prevalence misreported as numbers of cases per 100,000 (eg 10,000 cases in 100,000 thousand is 10%). But then it is often then misquoted as something like the prevalence was 10,000 cases per 100,000 case per year. Which is actually a cumulative prevalence over time and is more akin to incidence. Unfortunately such non standard ways of reporting incidence and prevalence only results in confusion. Thus it is advisable to report in a standardized way.

Comment

Modify: Fisher exact test using SPSS (for example) with a significance of P<0.05 (or similar)

Response

We used the statistical programme R, NOT SPSS, so it would be incorrect to change the text to SPSS. We have already cited R. However, we have inserted the P<0.05 as significance.

Comment

I can see you have deleted the links

Response

We have **not** deleted the links! The links are cited in the text and are given in the reference list.

Comment

There was a substantial decrease in the number of reported cases and deaths due to CE and AE in 2020, which is associated with the outbreak of Coronavirus. CITE THIS SENTENCE.

Response

This does not need a bibliographic citation to support this statement and indeed non is available. What is clear is from the data presented there was a substantial decrease in the numbers of reported cases of CE and AE in 2020. And in 2020 there was the outbreak of Coronavirus (COVID 19). We were hypothesising that the reason for the decrease in CE and AE reported cases was that the medical services were fully occupied dealing with the COVID 19 pandemic and hence it was not possible to investigate and treat as many suspected cases of echinococcosis. Therefore we have now replaced this sentence with the following text.

“There was a substantial decrease in the number of reported cases and deaths due to CE and AE in 2020 (Figure 1, Tables 1 and 2). This was in the same year as the start of the COVID 19 pandemic and hence this decrease in reported incidence of echinococcosis may be due to the health services having to manage the large numbers of COVID 19 patients and thus fewer resources were available to investigate and treat cases of suspected echinococcosis.”

We have also moved this text to the end of the paragraph where it would be more appropriate, especially since we moved other text to this paragraph in response to another comment (see below).

Comment

The high number of disability-adjusted life years – DALYs deaths demonstrates not only the deadliness of these diseases… Either remove “not only” or add “but also” followed by other confirmatory words.

Response

It is now linked to the following sentence so it now reads

“The high number of disability-adjusted life years – DALYs and deaths demonstrates not only the deadliness of these diseases but also indicate additional social and economic burdens on families and society as a whole.”

Comment

This paragraph is discussing the continuous increase in IR for CE and AE in the last two decades. It came after the DALY paragraph. It can be better incorporated in the second paragraph of the discussion which discusses the fatality and mortality.

Response

It seems the original comment on the first review had a mismatch between the line numbers where the text was. I now see the text the reviewer was referring to and have moved it accordingly.

Comment

This is not the reviewer’s job

Response

In the comment by the reviewer in the fist submission, the reviewer wrote the following: “Line 243: the public health challenges paragraphs are not benefiting from the study results and are

benefiting the discusses due to their general nature. General wording is just filling space.”

Unfortunately the English was not clear in this comment and we could not understand the reviewer’s point (I am a native English speaker). We asked, in response, for the reviewer to clarify the point (s)he was making, so the comment “not the reviewer’s job” is not helpful, and therefore it is still not possible to deal with this issue.

Reviewer 3

Comment

The presentation of the results is currently unacceptable for publication due to severe formatting errors. Table 4 is unintelligible in its current form, the headers contain unprofessional typographical errors (e.g., "Internationao", "doller") and the columns are misaligned, making it impossible to verify the economic data. Additionally, Table 1 lacks consistent decimal formatting (mixing commas and points).

Response

The typographical errors in Table 4 have been corrected. The manuscript was prepared in MS Word. In our version and the version we submitted the columns are properly aligned. However, this issue should have been noted in the proof version (pdf). I have since altered the formatting so that there is better alignment in the pdf.

In table 1, there is a single case of decimal formatting where a , is used as . This has now been corrected.

Comment

The conclusions are broadly supported by the data, but the clarity is undermined by inconsistent terminology. The authors interchange "mortality" (deaths per population) and "case fatality rate" (deaths per case) when comparing AE and CE. Precise terminology is required here to accurately convey the comparative severity of the diseases.

Response

There was a single occasion where mortality was used instead of case fatality rate. This has been corrected. But to avoid any further ambiguity, mortality has been replaced by deaths where appropriate.

Comment

While the authors have addressed several methodological queries raised in the first round, the revised manuscript is not in a publishable state. The authors stated that specific corrections were made, yet the manuscript file provided still contains the original errors. Furthermore, Tables 4 and 5 contain severe formatting errors and typos (e.g. "Internationao", "doller").

Response

Due an oversight, not all errors were corrected on the first revision. However, we hope that these typographical and formatting errors have now been corrected.
